# “This is how I want it”: Exploring the use of a workbook with persons with dementia to support advance care planning engagement

**DOI:** 10.1177/14713012221127358

**Published:** 2022-09-23

**Authors:** Tamara Sussman, Jack Lawrence, Rebecca Pimienta

**Affiliations:** School of Social Work, 150753 McGill University, Montreal, Canada

**Keywords:** advance care planning, self-directed tool, person with dementia, focus group, pilot intervention, end of life communication, palliative aproach

## Abstract

This mixed method sequential study reports focus group and pilot intervention findings that (1) explore the views of persons with dementia and their caregivers on using a self-directed advance care planning engagement workbook (*Your Conversation Starter Kit*) and (2) uncover the conditions that encouraged and hindered workbook use. In Phase 1, we conducted five focus groups consisting of 10 persons with dementia and eight family members/caregivers from two urban Canadian cities to explore overall impressions of the workbook and factors that might affect its use. In Phase 2, we empirically explored the factors identified in Phase 1 by distributing the workbook to 24 persons with dementia. The combined findings suggest that the workbook offers promise in supporting advance care planning engagement for persons with dementia in the early stages of their condition. Involving family/caregivers and clarifying some of the ranked questions might improve the workbook’s use. Persons with dementia without familial support or those who have never contemplated advance care planning may require additional guidance prior to workbook distribution.

## Introduction

Advance care planning is a process of reflecting on, discussing, and sometimes documenting values and preferences for future care in advance of need (Sudore et al., 2017). This iterative multistage process aims to center persons with dementia in their care by capturing values and preferences early in the trajectory of their condition, when capacity is most consistently present (Hirdes et al., 2011; Van der Steen, Radbruch, et al., 2014; Wendrich-van Dael et al., 2020).

Advance care planning can improve end-of-life (EOL) care for persons with dementia and their families by reducing the frequency of invasive interventions (Litzelman et al., 2017; Nicholas et al., 2014) and increasing the concordance between patients’ and families’ preferred EOL wishes (McMahan et al., 2021). Yet, less than 40% of persons with dementia across the globe are currently given the opportunity to participate in an advance care planning process (Sellars et al., 2019). Barriers that underly this low level of engagement include uncertainty regarding when advance care planning should be initiated and which topics should be discussed; a hesitation to engage in conversations about death and deterioration with persons with dementia; and limited health provider time and knowledge to direct the process (Giordano et al., 2022; Simon et al., 2015; Van der Steen, Van Soest-Poortvliet, et al., 2014; Wendrich-van Dael et al., 2020).

In recent years, interactive tools designed for self-use like workbooks, videos, and card games have been developed to address many of these barriers. These tools encourage readiness for advance care planning engagement (e.g., explain what makes advance care planning important), prompt value and preference clarification (e.g., ask questions to elicit personal preferences for future care), and support advance care planning communication (e.g. encourage discussions with family/caregivers and healthcare professionals) (Bridges et al., 2018; Sussman et al., 2020; Van Scoy et al., 2016). However, the acceptability of these resources for persons with dementia and their caregivers have yet to be examined.

The current mixed methods sequential study moves the literature forward by exploring the use of one publicly available advance care planning engagement workbook called Your Conversation Starter Kit (The Conversation Project & Institute for Healthcare Improvement, 2016). Specifically, the study aims to explore the views of persons with dementia and their caregivers on using the workbook to support advance care planning engagement. It further seeks to uncover the conditions that support and hinder workbook use.

## Your conversation starter kit

The workbook is a publicly available interactive 12-page workbook that is informed by a staged model of advance care planning engagement. The four steps included in the workbook are designed to move people through a process of personal reflection and communication (Sussman, Kaasalainen, et al., 2021). Each section contains open-ended ended questions, while the second section also contains ranked choice questions. The first two steps (Get Ready & Get Set) promote reflection on how to engage with a family member/caregiver in advance care planning discussions through tips, prompts, and questions that focus on values, concerns, and preferences for future care. The last two steps (Go & Keep Going) direct users towards discussions with family/caregivers. This layout is aligned with advance care planning frameworks structured around behavioural change, which posit reflection as an individual process that a person with dementia takes in preparation for advance care planning actions, such as discussions with family/caregivers and preference documentation (McMahan et al., 2013; Sudore et al., 2008). The workbook was developed by the Institute for Healthcare Improvement as a part of a public engagement initiative to improve advance care planning awareness and uptake for all adults.

We selected this tool because of its relevance to persons with dementia, as it prompts to reflection on what constitutes quality of life and quality of care, family/caregiver involvement in decision-making, and appropriate moments for advance care planning conversations (Giordano et al., 2022; Sussman et al., 2020; Sussman, Kaasalainen, et al., 2021; Van der Steen, Van Soest-Poortvliet, et al., 2014). Further, the generic nature of the tool does not necessitate that persons with dementia accept their diagnosis prior to use, thus overcoming an obstacle to advance care planning conversations identified by family/caregivers and health providers (Hirschman et al., 2008). Finally, the tool has already been pilot tested for use with older persons living with frailty (Kaasalainen et al., 2020; Lum et al., 2020).

## Study design

This study used an exploratory sequential mixed methods design to meet its aims (Creswell, 2013). An exploratory sequential design involves collecting and analysing qualitative and quantitative data in two consecutive phases. The design is considered particularly useful for informing intervention protocols such as that employed in the current study (Creswell & Plano Clark, 2011).

In Phase 1, we used focus groups of persons with dementia and their caregivers to gauge the overall acceptability of the workbook and identify the factors that might be associated with its use. In Phase 2, we distributed the workbook to persons with dementia and their caregivers, and examined the factors associated with its use identified in Phase 1. The workbook responses of participating persons with dementia were tabulated to determine how its components were used.

Purposive sampling methods were used to recruit participants for both study phases. In Phase 1, persons with dementia and their caregivers were recruited from two Alzheimer Society chapters in Canada (one in Quebec and one in Ontario). In Phase 2, participants were recruited from the same Alzheimer Society chapters as well as a home care department in Quebec. In both phases, staff within the recruiting organizations identified potential participants, provided preliminary information about the study and, if potential participants were agreeable, passed on relevant contact information to the study research coordinator. All interested potential participants were then contacted by the research coordinator, who reviewed the study’s purpose and details of involvement, emphasizing that participation was voluntary and would have no impact on their organization’s service provision.

Staff recruiting participants in both phases were asked to reach out to potential participants who were (a) French or English speaking (b) clinically judged by staff to be a person with dementia in the early stages of their condition and capable of providing consent to participate in advance care planning discussions, or (c) an informal caregiver supporting a person with dementia capable of participation. Former research has suggested that simply participating in focus group deliberations about advance care planning can activate contemplation (Dube et al., 2021; Sussman, Pimienta, & Hayward, 2021). Hence, in order to avoid confounding results on workbook use and advance care planning activation, Phase 2 recruitment targeted participants who had not participated in Phase 1.

Signed written consent was obtained from participating persons with dementia and caregivers in both phases. In Phase 1, written consent was obtained on the day of focus group deliberations. In Phase 2, written consent was obtained on the day of workbook distribution. Willingness and capacity to participate was monitored by members of the research team throughout the duration of the study. All research team members involved in data collection in both phases were social work and nursing graduate students with training in dementia care (Wilson, 2011). All enrolled participants were sent a $10.00 gift card as a token of appreciation for their participation.

The research was conducted in accordance with the standards of the Tri-Council Policy Statement for Ethical Conduct for Research Involving Humans (Canadian Institutes of Health Research et al., 2018) and was approved by the Research Ethics Board Office at McGill & McMaster University.

The next section will report on Phase 1’s methods, analysis, and results. The following section will report on Phase 2’s methods, analysis, and results.

## Phase 1: Focus groups

### Data collection

Focus groups were held for approximately 60–90 minutes and were facilitated by two members of the research team. Both facilitators had expertise in active listening, group facilitation, and communication with persons with dementia, and were well-positioned to ensure the engagement and comfort of all participants.

A semi-structured interview guide was used to explore participants’ views on the acceptability of the workbook. The guide sought to elicit overall impressions of the workbook (e.g. ‘what do you think about the idea of completing a workbook like this?), thoughts on timing of workbook distribution and completion (e.g. ‘when is a good time receive/complete a workbook like this?’), and ideas about who should be involved in workbook completion (e.g. ‘who do you think should complete this workbook?’ and ‘when, if at all, should family/caregivers and health providers be involved?’). The guide also sought to elicit participants’ overall experiences with and perceptions of advance care planning; those findings are reported elsewhere (Sussman et al., 2020).

While our original design separated persons with dementia and caregivers to create a safe space for each group’s voices to be heard (Wiersma et al., 2016), some persons with dementia expressed an interest in participating with their caregiver. Hence, some mixed groups were offered to accommodate this preference. All focus group participants completed a short questionnaire that asked their age, gender identity, and the length of time they had been living with or supporting someone living with dementia. Caregiving participants were asked how they were related to the person with dementia (e.g., as a spouse, partner, child, friend).

### Data analysis

A four-step semantic thematic analysis was used to analyze the focus group discussion transcripts (Braun & Clarke, 2019). In step 1, all text excerpts related to the workbook were extracted and reviewed by [the third author – RP] to gain familiarity with the data. In step two, [the first and third author – TS] discussed [the first author’s] preliminary impressions and together created a set of descriptive codes thought to broadly capture the sentiments expressed by study participants related to workbook acceptability and use. Preliminary descriptive codes developed at this second stage included: *perceived benefits of using the workbook*, *perceived challenges of using the workbook*, *reactions to the workbook content*, and *recommendations for workbook implementation*.

In step three, all excerpts and their associated codes were reviewed independently and then together by [the first and third authors – TS and RP] with the aim of generating descriptive themes thought to capture the essence of participants’ deliberations (Braun & Clarke, 2019). This process involved reflection on and discussion of possible meanings and patterns within, between, and across codes. For example, we noted that extracts coded as *perceived benefits of the workbook* and *recommendations for workbook implementation* appeared to represent examples of a larger theme of the workbook’s potential utility for different stages of the advance care planning process. We hence developed the descriptive theme *the workbook supports initial and ongoing advance care planning engagement* to better represent this idea. This process also illuminated some differences between persons with dementia and their caregivers around preferred use. These divergent perspectives were captured in the theme *the workbook* can *be used alone or with others.*

In the fourth step of analysis, all selected extracts and their associated themes were reviewed for accuracy, comprehensiveness, and redundancy (Braun & Clarke, 2019). This process involved comparing and contrasting themes and their associated extracts with the original un-coded transcripts. No new themes emerged from this re-examination suggesting thematic saturation (Saunders et al., 2018). At this stage, all coded French extracts were translated by [third author – RP] and verified for accuracy by [first author – TS]. The team’s capacity to work with French transcripts until the final stage of analysis aligns with recommendations in the literature, as it helps to preserve the contextual meanings of extracted text (Roth, 2013).

### Participants

A total of 18 participants, including 10 persons with dementia and eight family members/caregivers, participated in five focus groups. One focus group was conducted with persons with dementia only (Persons with Dementia Group), one focus group was conducted with family/caregivers only (Caregiver Group), and three focus groups combined persons with dementia and family/caregivers (Mixed Groups 1, 2, and 3). Focus groups ranged from two to six participants, and the mean group size was four participants.

Persons with dementia ranged in age from 50 to 84 years (M = 71, SD = 9). Eight persons with dementia were men, and most had been living with their diagnosis for 5 years or less. Family caregivers ranged in age from 64 to 85 years (M = 74, SD = 7) and were predominantly women (six of eight) and spouses (six of eight).

All focus group participants have been ascribed pseudonyms to maintain confidentiality.

### Overview of focus group findings

Focus group deliberations suggested that both persons with dementia and their caregivers found the workbook helpful because it could serve to *“take the stress away”* (Margaret, caregiver, Mixed Group 1) and ensure that “*there’s no guessing*” (Bill, person with dementia, Mixed Group 1). Deliberations further revealed that the content of the workbook was perceived to be “*non-threatening”* (Marie, caregiver, Mixed Group 2) and *“useful”* (Geneviève, person with dementia, Mixed Group 3) intimating that both persons with dementia and caregivers viewed the workbook as a viable option for supporting advance care planning engagement.

The three themes presented below offer further insight into the factors thought to support workbook use and the elements of the workbook viewed to be of relevance and utility to persons with dementia and their caregivers. Differences between the views expressed by persons with dementia and caregivers, when evident, are also noted within the body of each theme.

### Theme 1: The workbook can be used alone or with others

Participants across focus groups agreed that the workbook was both useable and useful. However, analysis of the findings suggested that persons with dementia and their caregivers held some divergent perspectives on how the workbook might optimally be used. Some persons with dementia felt that because the workbook appeared to elicit highly personal reflections, it would be optimally used alone prior to engaging in conversations or communication with others. This desired form of use was expressed by two persons with dementia as follows:

”I would fill it out then I would talk about it with the people who are important.” (Jean, person with dementia, Persons with Dementia Group)

[pointing to the workbook] “I’d say, ‘There it is …. this is how I want it.’” (Geneviève, person with dementia, Mixed Group 3)

Conversely, family/caregivers seemed to express the sentiment that the workbook could be used collaboratively from the start. According to family/caregivers, using the workbook in this way would allow for reflections and dialogue to co-occur. Marie expressed this preference as follows:

I think you could sit down now, you'd have to be in the right frame and that, but there's no threat. It's easy-going and you could have a conversation going back and forth… I think that would be great to have it as a family (Marie, caregiver, Mixed Group 2)

Not all participants expressed certainty regarding their preference for initial workbook use. However, our analysis suggested that persons with dementia tended to favour the idea of using the workbook alone, while caregivers appeared to favour their involvement from the outset.

### Theme 2: Workbook supports initial and ongoing advance care planning engagement

Although many participants agreed that conversations about future care can be intimidating, comments across groups suggested that the workbook had potential to initiate and support advance care planning engagement.

Participants who had no prior engagement with advance care planning felt the workbook could be used to “*open the conversation”* (Geneviève, person with dementia, Mixed Group 3) because the content *“gives ideas”* (André, person with dementia, Persons with Dementia Group) and *‘good prompts’* (Ruth, caregiver, Caregiver Group) for what to think about and discuss. The direction offered by the workbook was viewed as useful for initial advance care planning engagement because people “*don’t always know what to [think about] because [they] have not faced it yet*” (Pierre, person with dementia, Persons with Dementia Group).

Participants who had prior experience with advance care planning also saw utility in the workbook because they felt it could reinforce, maintain, or elaborate on former future care discussions and reflections. This view is depicted in the following exchange between family/caregivers who had already begun advance care planning conversations:

*Kim: “I think it’s great ‘cause I’d probably find something in there that I haven’t done.”*

Marie: “Exactly. Yeah. The stuff you didn’t think of.”

*Kim: “And I always thought, it can’t hurt, the more I can do.”*

(person with dementia and caregiver, Mixed Group 3)

While participants within and across focus groups differed in their prior engagement in advance care planning, all appeared to consider the workbook useful for their circumstances suggesting that the workbook may be acceptable for various stages in the advance care planning process.

### Theme 3: interactive elements and probes about decisional involvement viewed as useful

Both persons with dementia and family/caregivers agreed that the interactive components of the workbook were useful as *“writing things down can open up a conversation”* (Bonnie, caregiver Mixed Group 1).

Persons with dementia appeared particularly drawn to the ranked questions in the workbook, nodding in agreement when André stated, *“I like the string of [ranked] questions a lot, the first questions [about] knowing the details of my condition and my [preferred] involvement in treatment decisions.”* (André, person with dementia, Persons with Dementia Group).

No participants expressed any concerns about the content of the workbook suggesting that the content of the workbook was acceptable to both persons with dementia and caregivers.

## Phase 2: Workbook distribution and use

### Data collection

On the date scheduled for workbook distribution, a member of the research team met with all dyads (those who enrolled with a family member/caregiver) and solo participants (those who enrolled alone); provided a brief overview of advance care planning and its purpose; and distributed the workbook for self-use. While participants were given the choice to meet the researchers at the participating university, their own home, or another location of their choice (e.g., a local library), all participants elected to receive a home visit.

Two weeks after the distribution of materials, the research team member who had conducted the initial meeting checked the status of workbook completion by calling the enrolled person with dementia and/or their caregiver. If the workbook was completed at that time, a follow up visit was scheduled to collect completed workbooks. If participants had not completed the workbook but expressed a desire to do so, an additional 2 weeks was granted, at which time a follow up visit was scheduled to collect the workbook. Collected workbooks were photocopied and then returned by mail.

### Predictor measures

#### Participant profiles

At the time of workbook distribution, all participating persons with dementia and family/caregivers completed a short questionnaire that asked their age, gender, marital status, level of education, and the length of time they had been living with or supporting someone living with dementia. Caregivers were also asked to identify their relationship to their loved one with dementia. This demographic information was used to provide an overview of the Phase 2 sample and explore the relationship between variables found to affect advance care planning engagement in the literature (e.g. gender, level of education) (Carr & Khodyakov, 2007).

#### Solo versus dyadic use

Our focus group deliberations noted divergent preferences in workbook use between persons with dementia and caregivers. We therefore intimated whether participants enrolled alone or with a caregiver, to explore potential differences between solo and dyadic use.

#### Prior advance care planning engagement

Our focus group participants felt the workbook could both activate and reinforce advance care planning engagement. To explore if persons with different levels of prior advance care planning engagement would use the workbook, we asked participants to complete the 24-item advance care planning engagement survey at the time of workbook distribution (Sudore et al., 2013). Responses to all items were ranked on a Likert scale ranging from (1), indicating low engagement on a given item, to (5), indicating high engagement. The survey has been validated on a sample of older adults with strong internal consistency (Cronbach’s alpha, 0.94) and test-retest reliability (interclass correlation, 0.70) (Sudore et al., 2013) and has previously been used to measure advance care planning engagement amongst persons with dementia (Kotwal et al., 2021; Vellani et al., 2022).

### Outcome measures

#### Workbook use

We measured workbook use in two ways. First, all workbooks returned with written entries to the interactive elements were considered *used*. Workbooks returned with no written entries, or not returned at all, were considered *unused*. Second, the four sections of each workbook were reviewed to identify which were used in-whole or in-part. Interactive elements with written entries were considered *used*, while interactive elements without written entries were considered *unused*.

### Data analysis

Participant profile questionnaires, overall advance care planning engagement scores, and coded workbook responses were descriptively analyzed using means and standard deviations for continuous data and frequencies and percentages for categorical data. These analyses provided an overview of participant profiles, pre-intervention levels of advance care planning engagement, and an indicator of workbook use.

We explored differences between users and non-users across overall advance care planning engagement survey scores using an independent 2-tailed t test. All bivariate analyses using overall use/non-use as an outcome measure were conducted using Fishers exact tests.

Ranked workbook question responses were coded as *clear* if they endorsed a scale’s extreme end (i.e., a ranking of 1, 2, 4, or 5), and *unclear* if they endorsed the middle of the scale (i.e., a ranking of 3). Both *clear* and *unclear* ranked responses, as well as responses to the workbook’s open-ended questions, were tabulated and presented with percentages.

### Results

#### Participant profiles

Twenty-four persons with dementia were recruited from three sites. Persons with dementia ranged in age from 61 to 88 (M = 78, SD = 7), and 13/24 (54%) were men. While we initially aimed to recruit a distinct sample for Phase 2, one person who had participated in Phase 1 expressed an interest in participating, and we felt it was not ethically justified to exclude them. On average, persons with dementia had been connected to the Alzheimer Society for 2.4 ± 2.5 years. Most persons with dementia were married (18/24, 75%) and had attained some level of post-secondary education (14/24, 58%).

Seventeen of the 24 persons with dementia enrolled with an informal caregiver. Caregivers ranged in age from 53 to 81 (M = 69, SD = 10) and 13/17 (76%) were women. Caregivers were most commonly the spouse of persons with dementia (13/17, 76%).

The average advance care planning engagement score was 3.94 (SD = 0.67), which is suggestive of moderate advance care planning engagement (Sudore et al., 2013).

#### Factors associated with workbook use

Fisher exact tests revealed that those enrolled with a family member/caregiver (17/24, 71%) and those who were married (18/24, 75%) were more likely to complete a workbook than those who were enrolled alone (7/24, 29%; *p* = .0088) and not married (6/24, 25%; *p* = .0501). There were no significant differences in workbook use between gender (*p* = .0953) and education level (*p* = .6785). Of persons with dementia enrolled with a caregiver (n = 17), there was a significant relationship between the caregiver’s role and workbook usership (*p* = .0223). Almost all persons with dementia paired with their spouse used the workbook (92%, 12/13) whereas only 25% (1/4) of persons with dementia paired with their children used the workbook. These results are summarized in Table 1.

**Table 1.** Factors associated with workbook use.

		User	Non-user		*p Value*
Persons with dementia	Advance care planning engagement	4.33 ± 0.54	3.40 ± 0.43		< 0.01
	Men	10	3	13	0.0953
	Women	4	7	11	
		14	10	24	
	Paired	13	4	17	< 0.01
	Solo	1	6	7	
		14	10	24	
	> High school	9	5	14	0.6785
	≤ High school	5	5	10	
		14	10	24	
Family	Spouse	12	1	13	0.0223
	Child	1	3	4	
		13	4	17	

Contrary to the impressions of focus group participants, workbook users (M = 4.33, SD = 0.537) were more likely to have engaged in some form of advance care planning prior to workbook use than Non-Users (M = 3.40, SD = 0.427), t (22) = 4.551, *p* < .01.

#### Overview of workbook use

Fourteen of the 24 persons with dementia (58%) enrolled in the study returned completed workbooks. Table 2 provides an overview of the extent to which the open questions were used by workbook step (i.e., section). Nine of the 14 workbook users (64%) used at least one of the two open-ended questions in Step 1 and 13 of 14 users (100%) completed at least one of the five open questions in Step 2. Examples of responses to these questions revealed clear values and wishes like “*as long as* *I am capable of living without pain, I would not want to hasten my death*” (Leonard, 83) and “[*I wish] a volunteer [would] play his violin at the funeral home*” (Mable, 88). Nine of 14 users (64%) used Step 3’s one open-ended question while only six of 14 users (43%) completed at least one of the three open-ended questions in Step 4. Recorded responses in Step 4 suggested that those who had reflected on or discussed their wishes with others were satisfied with their engagement. Comments included “I *have the perception that the whole family (my children and spouse) have understood and agree with my wishes*” (Winston, 71) and “*I feel good about the whole process and my thinking*” (Hanz, 73).

**Table 2.** Use of the workbook’s opened-ended questions.

Step	Open-ended questions	Used	Example
**1**	What do you need to think about or do before you feel ready to have the conversation?	9 (64%)	“How willing my family [is] to be responsive to having this conversation. How open and honest I am willing to be.” (Charles, 84)
	Do you have any particular concerns that you want to be sure to talk about?	9 (64%)	“All my documents have already been done, but I often wonder when I should actually do them (i.e., at a more advanced stage, serious illness, cancer)” (Hanz, 73)
**2**	What matters to me at the end of life is…	11 (79%)	“It would be a big gift to still have my lucidity [and have] my two sons on either side of me” (Daisy, 88)
	What kind of role do you want to have in the decision-making process?	12 (86%)	“If I am able, I would like to express what I think” (Winston, 71)
	What do you notice about the kind of care you want to receive?	11 (79%)	“I would like to be in the place that will be the least demanding for the loved ones around me, especially my wife. For example, to be in a hospice where there are staff” (Hanz, 73)
	What role do you want your [family/caregivers] to play?	12 (86%)	“If there was a volunteer who could play his violin, I would ask for special permission to hear him” (Mable, 88)
	What do you feel are the three most important […] wishes and preferences for end-of-life care?	13 (93%)	“No excessive treatment measures; adequate care; maintain my comfort zone” (Lionel, 75)
**3**	What do you want to be sure to say?	9 (64%)	“My quality of life is more important than longevity. I want to be relieved by medication even if it means shortening my days. I hope that my loved ones will be there.” (Winston, 71)
**4**	Is there something you need to clarify that you feel was misunderstood or misinterpreted?	5 (36%)	“Does help in dying exist?” (Lionel, 75)
	Who do you want to talk to next time?	6 (43%)	“No one” (Ethel, 94)
	How did this conversation make you feel?	5 (36%)	“Very serene and the conversations took place in harmony” (Winston, 71)
	What do you want to make sure to ask or talk about next time?	5 (36%)	“Reread this document… and learn to detach and let go” (Hanz, 73)

Table 3 provides an overview of Step 2’s ranked questions. All respondents answered all ranked questions. All or most respondents were clear about the amount of information they wished to have about their condition (14/14, 100%), time until death, the importance of quality versus quantity and preferences for information sharing with family/caregivers (13/14, 93% for each). Many respondents were unclear about whether they were concerned about getting too little versus too much care in the future (9/14, 64%) and the extent to which they wanted to share decision-making with family/caregivers and health professionals (6/14, 43%).

**Table 3.** Use of the workbook’s ranked questions.

Ranking^a^		Clear^b^	Unclear^c^	Missing
	***As a patient, I’d like to know:***	14 (100%)	0 (0%)	0 (0%)
1	Only the basics about my condition and my treatment			
5	All the details about my condition and treatment			
	***As doctors treat me, I would like:***	8 (57%)	6 (43%)	0 (0%)
1	My doctors to do what they think is best			
5	To have a say in every decision			
	***If I were in the terminal illness, I would prefer to:***	13 (93%)	1 (7%)	0 (0%)
1	Not know how quickly it is progressing			
5	Know my doctor’s best estimation for how long I have to live			
	***How long do you want to receive medical care?***	13 (93%)	1 (7%)	0 (0%)
1	Indefinitely, no matter how uncomfortable treatments are			
5	Quality of life is more important to me than quantity			
	***What are your concerns about treatment?***	4 (29%)	9 (64%)	1 (7%)
1	I’m worried that I won’t get enough care			
5	I’m worried that I’ll get overly aggressive care			
	***What are your preferences about where you want to be during your last days?***	10 (71%)	3 (21%)	1 (7%)
1	I wouldn’t mind spending my last days in a health care facility			
5	I want to spend my last days at home			
**5**	***How involved do you want your*** *[****family/caregivers] to be in care decisions?***	8 (57%)	6 (43%)	0 (0%)
	I want [them] to do exactly what I’ve said, even if it makes them uncomfortable			
	I want [them] to do what brings them peace, even if it goes against what I’ve said			
	***When it comes to your privacy:***	11 (79%)	3 (21%)	0 (0%)
1	I want to be alone when the time comes			
5	I want to be surrounded by my family members or friends			
	***When it comes to sharing information:***	13 (93%)	1 (7%)	0 (0%)
1	I don’t want my [family/caregivers] to know everything about my health			
5	I am comfortable with [family/caregivers] knowing everything about my health			

^a^The workbook includes nine 5-point ranked questions, where responses of ‘1’ and ‘5’ represent opposite ends of a continuum.

^b^Frequency of non- ‘3’ responses (i.e., a response of 1, 2, 4, or 5), clearly endorsing one end of a continuum.

^c^Frequency of ‘3’ responses.

## Discussion

Advance care planning is an important aspect of early care for persons with dementia as it allows them to express their future care wishes and concerns while they are still capable of doing so. To support broader uptake, self-directed advance care planning tools like the *Your Conversation Starter Kit* workbook have emerged. Our study explored how the workbook could be used by persons with dementia and their family/caregivers in community settings, addressing a gap in the literature on advance care planning barriers and facilitators amongst persons with dementia (Giordano et al., 2022). Overall, our combined qualitative and quantitative findings suggest that the workbook is acceptable to persons with dementia and families/caregivers, who generally found the workbook useful and non-threatening. Our findings also illuminate important insights regarding workbook distribution that may improve its use.

The process of advance care planning has been conceptualized in stages of behavioural change (Sudore et al., 2008). In order to facilitate broader uptake of advance care planning, self-directed tools like the workbook used in this study can direct persons with dementia through each stage and its associated tasks. In *precontemplation*, the first stage, persons with dementia do not recognize advance care planning as relevant. Here, education about the importance of advance care planning and its benefits is recommended (Fried et al., 2016). The workbook meets this recommendation through the provision of a fact sheet (e.g., “90% of people say that talking with their loved ones about EOL care is important [but only] 27% have actually done so”) (The Conversation Project & Institute for Healthcare Improvement, 2016, p. 2). In *contemplation*, the second stage, persons with dementia begin an ongoing process of iterative reflection and value clarification. The workbook supports this stage through tips, prompts, and questions that focus on values, concerns, and preferences for future care. The later sections of the workbook are designed to move persons with dementia through the *preparation* and *action* stages of advance care planning, as they direct users towards preparing for and having discussions about future care with family/caregivers. Hence, this behavioural change model and workbook design positions reflection as an individual process that a person undertakes in preparation for advance care planning discussions with family/caregivers (McMahan et al., 2013, 2021; Sudore et al., 2013; Sussman et al., 2020).

In alignment with behavioural change models and the workbook’s design, focus group deliberations suggested that persons with dementia may prefer to use the workbook alone at first before engaging caregivers in a discussion about possible preferences and wishes. Conversely, caregivers expressed a preference for their involvement from the outset, seeing the reflective stage of advance care planning as an opportunity to engage in dialogue that can unravel preferences and wishes for future care. Indeed, some research lends support to this dyadic approach, suggesting that the context of supportive relationships dissipates the threat of thinking about futures and hence should be activated prior to encouraging these reflections (Fried et al., 2017; Sudore & Fried, 2010).

Our analysis of workbook distribution revealed that persons with dementia who enrolled with a family member/caregiver were significantly more likely to use the workbook than those enrolling alone. While we cannot speak to how and when persons with dementia engaged their caregivers in workbook use, this finding supports the dyadic approaches to advance care planning engagement endorsed by caregivers during focus group deliberations.

Regarding the timing of advance care planning engagement, our focus groups deliberations suggested that persons with varying levels of prior advance care planning engagement saw value in the workbook. For those who had not engaged in any advance care planning contemplation, the workbook was seen as an opportunity to express what they may want to have happen in the event their health declines. For those who had engaged in prior reflections or discussions, the workbook was seen as an opportunity to revisit prior conversations and clarify preferences and wishes.

However, our pilot distribution of workbooks intimated that those persons with dementia with prior advance care planning engagement were more likely to use the workbook than those with no prior advance care planning engagement. Hence, while the workbook may be useful for different stages of the advance care planning process, those at the precontemplative stages of advance care planning may be less likely to use it without support or encouragement.

Notably, our quantitative analysis of workbook distribution suggested education level was not associated with workbook use. This finding stands in contrast to other research examining the utility of informational tools and materials distributed to older persons experiencing frailty (Cutilli, 2007; Sussman et al., 2017). That the workbook did not appear to deter those with divergent levels of education from its use implies that its content is acceptable for persons with dementia of all educational backgrounds.

Our focus group deliberations suggested that both the substance and the style of the workbook questions were clear, useful, and non-threatening. Focus groups discussions further indicated that persons with dementia saw particular value in the ranked questions located in Step 2 of the workbook. Analysis of our workbook distribution affirmed and extended these sentiments. All elements of the workbook were used by many, and responses to open-ended questions in all four steps of the workbook generated many clear and actionable preferences that could be easily communicated to others, such as “*if there was a volunteer who could play his violin, I would ask for special permission to hear him*” (Mable, 88). However, while the ranked questions were the only component of the workbook used by all participants (lending some support that this style of questioning was effective), our analysis of workbook responses suggested that three of the ranked questions were more likely to generate unclear middle range rankings than the remaining six. Specifically, the ranked questions meant to elicit the extent to which persons with dementia wanted decisional involvement with physicians and family/caregivers, or had worries about overly aggressive future care, were most likely to generate unclear middle range responses. Given that decisional involvement and levels of medical intervention are of particular importance to persons with dementia and their family/caregivers, the wording of these questions should be improved and clarifying follow-up discussions should be encouraged (Bhatt et al., 2020; Menne & Whitlatch, 2007; Mitchell et al., 2017).

### Implications and recommendations

Our combined findings offer important implications for the timing and method of workbook distribution most likely to support use for persons with dementia. First, it may be most useful to distribute the workbook to persons with dementia in the presence of family/caregivers who are positioned to encourage advance care planning reflection and dialogue. Upon distribution, it could be emphasized that the earlier reflective steps in the workbook (which are worded as if they should be completed individually) can also be completed with family/caregivers to stimulate an iterative process of contemplation and discussion.

Second, persons with dementia who are at the pre-contemplative stage of advance care planning engagement (i.e., have not heard of nor thought about advance care planning) may benefit from staff-directed guidance prior to receiving the workbook. A prior advance care planning engagement survey, such as the one used for this study, could be used to assess whether guidance is required (Sudore et al., 2013). Simply asking persons with dementia if they have spent any time thinking about or discussing their future care concerns could be another way to assess the appropriateness of distributing the workbook without preliminary guidance.

Third, the potential for the workbook to activate actionable reflections may be strengthened if some of the ranked questions are reworded to improve clarity. For example, the question on treatment concerns is double-barrelled (Menold, 2020) because it asks users to choose between two worries — that they ‘won’t get enough care’ (a ranking of 1) or they ‘will get overly aggressive care’ (a ranking of 5) — disregarding the possibility that one could be equally concerned about both worries. Other double-barrelled questions ask users to select between preferences for (1) ‘doctors to do what is best or (5) ‘having a say in every decision’, and for (1) family/caregivers ‘to do exactly’ what the persons with dementia said they wanted ‘even if it makes them uncomfortable’ or (5) family/caregivers ‘to do what brings them peace’ even if it contrary to what the person with dementia instructed them to do. These questions could also be clarified by prompting users to explain their middle range responses through discussions with staff or family/caregivers.

Fourth, the pattern of workbook use in our study illuminates the need to consider persons with dementia who do not have the support of family/caregivers. It is unlikely that these persons with dementia will benefit from reliance on self-directed advance care planning workbooks without support of some kind (Piers et al., 2018; Wendrich-van Dael et al., 2020). Approximately 13% of persons with dementia in North America reside alone, many of whom have limited access to familial support (Gould et al., 2015). This isolation is further complicated for persons with dementia who have intersecting minoritized identities, like sexual orientation and HIV status (Dube et al., 2021) and psychiatric disorders (Donovan & Blazer, 2020), as they face active and systemic exclusion from accessing equitable palliative care (Rosa et al., 2022). In such cases, sustained efforts to foster connections with health providers or community agencies (e.g., Alzheimer Societies) appear necessary prior to distributing an advance care planning engagement workbook like the one used in this study. Studying how self-directed materials can best support advance care planning engagement for persons with dementia with social vulnerabilities is an important area of future research and practice.

Taken together, our findings suggest that self-directed workbooks hold promise for engaging persons with dementia in advance care planning. However, if distribution remains solely untargeted (i.e., publicly available on a website for those interested), it is unlikely to be used by many persons with dementia. We therefore suggest that organizations supporting persons with dementia consider distributing a workbook like the *Your Conversation Starter Kit* and facilitating its use under certain circumstances.

During the time of writing, a revised version of the workbook was developed (Your Conversation Starter Guide)*.* While the general format and length has been retained, some additional questions related to quality of life have been added. Worries related to quality of life are often expressed by persons with dementia, who fear a future that could threaten their capacity to experience joy and meaning (Sussman, Pimienta, & Hayward, 2021). We therefore believe these workbook additions make the tool that much more suitable for persons with dementia.

### Study limitations

This study should be viewed in light of two important limitations. First, while pilot interventions in the literature have used comparably small sample sizes (Ahluwalia et al., 2021; Miller et al., 2019), our small sample size precluded us from examining divergent uptake of the workbook based on factors shown to impact advance care planning engagement, such as race and ethnicity (McAfee et al., 2019; Pettigrew et al., 2020). Second, both phases of our study relied on service providers reaching out to and recruiting persons with dementia and their caregivers. As a result, we do not know how many individuals were approached but declined to participate, posing a potential threat to the transferability of study findings (Phase 1) and the generalizability of results (Phase 2).
